# Can the function of the tubarial glands be evaluated using [^99m^Tc]pertechnetate SPECT/CT, [^18^F]FDG PET/CT, and [^11^C]methionine PET/CT?

**DOI:** 10.1186/s13550-021-00779-6

**Published:** 2021-03-31

**Authors:** Yohji Matsusaka, Tomohiko Yamane, Kenji Fukushima, Akira Seto, Ichiro Matsunari, Ichiei Kuji

**Affiliations:** 1 Department of Nuclear Medicine, Saitama Medical University International Medical Center, 1397-1 Yamane, Hidaka, Saitama, 350-1298 Japan; 2 Division of Nuclear Medicine, Department of Radiology, Saitama Medical University Hospital, 38 Moro-Hongo, Moroyama, 350-0495 Japan

**Keywords:** Tubarial gland, Salivary gland, Tonsil, [^99m^Tc]pertechnetate, [^18^F]fluorodeoxyglucose, [^11^C]methionine

## Abstract

**Background:**

The tubarial glands (TGs) are recently reported as newly found salivary gland structures that can be organs at risk predominantly localized in the tori tubarius in the nasopharynx using prostate-specific membrane antigen positron emission tomography/computed tomography (PSMA PET/CT). The aims of this study were to analyze uptake in the TGs compared with that in the other salivary glands and palatine tonsils using [^99m^Tc]pertechnetate SPECT/CT, [^18^F]FDG PET/CT, and [^11^C]methionine PET/CT and to confirm whether these three imaging modalities are useful in evaluating the physiological function of the TGs. Twelve and 130 patients, who underwent [^99m^Tc]pertechnetate SPECT/CT and [^18^F]FDG/[^11^C]methionine PET/CT, respectively, were retrospectively included. [^99m^Tc]pertechnetate uptake in the tori tubarius was visually assessed and semiquantitatively compared with that in the background, parotid salivary glands (PSGs), submandibular salivary glands (SmSGs), and sublingual salivary glands (SlSGs). Correlations of [^18^F]FDG and [^11^C]methionine uptakes in the tori tubarius with those in the other three salivary glands and palatine tonsils were analyzed.

**Results:**

[^99m^Tc]pertechnetate uptake in the tori tubarius was invisible and was not significantly higher than that in the background. Both [^18^F]FDG and [^11^C]methionine uptakes in the tori tubarius were correlated with that in the palatine tonsils (*r* = 0.56, *p* < 0.0001; *r* = 0.48, *p* < 0.0001, respectively). [^18^F]FDG uptake in the tori tubarius was not positively correlated with that in the PSGs, SmSGs, and SlSGs (*r* = − 0.19, *p* = 0.03; *r* = − 0.02, *p* = 0.81; *r* = 0.12, *p* = 0.17, respectively). [^11^C]methionine uptake in the tori tubarius was correlated with that in the SmSGs and SlSGs (*r* = 0.24, *p* = 0.01; *r* = 0.32, *p* < 0.01, respectively), but not with that in the PSGs (*r* = 0.16, *p* = 0.08).

**Conclusions:**

The TGs were undetectable on [^99m^Tc]pertechnetate SPECT/CT. Both [^18^F]FDG and [^11^C]methionine uptakes in the tori tubarius were clearly affected by that in the palatine tonsils and was little related to that in the other salivary glands. Therefore, it seems difficult to evaluate the physiological function of the TGs as salivary glands using [^99m^Tc]pertechnetate SPECT/CT, [^18^F]FDG PET/CT, and [^11^C]methionine PET/CT imaging.

## Background

The tubarial glands (TGs) are recently reported by Valstar et al. as newly found salivary gland structures that can be organs at risk localized in the posterior wall of the nasopharynx using prostate-specific membrane antigen positron emission tomography/computed tomography (PSMA PET/CT) [1]. This report has led to widespread media attention [2]. Nuclear medicine physicians know well that PSMA ligands strongly accumulate in the salivary glands [3]. Although some researchers noticed that strong uptake of PSMA ligands was seen not only in the salivary glands but also in the dorsal wall of the nasopharynx [4], Valstar et al. investigated the TGs histologically and clinically in detail and named the gland TGs for the first time. They reported that irradiation of the TGs was associated with adverse effects, such as xerostomia and dysphagia. However, the physiological and clinical importance of the TGs has not yet been clarified [5].

The main physiological function of the salivary glands is to secrete saliva containing electrolytes, mucin, and proteins. Although uptake of PSMA ligands in the TGs seems to be useful for evaluating their existence or volume, the uptake is not appropriate to directly evaluate the secretory ability of the TGs, because PSMA ligands are not released from the salivary glands even after stimulation with vitamin C [6]. Therefore, the objective methods, other than PSMA PET/CT, which enable the evaluation of TG’s secretory ability are warranted to clarify the pathophysiology of the diseases involving the TGs or to diagnose them.

[^99m^Tc]pertechnetate scintigraphy has been widely used for the clinical evaluation of the salivary gland function. [^99m^Tc]pertechnetate ([^99m^Tc]TcO_4_-) acts as an electrolyte tracer in such examinations. Dynamic scans on anterior–posterior planar images have high time resolution, and the use of vitamin C stress test enables the evaluation of both accumulation in and secretion from the salivary glands [7, 8]. However, since planar images are two-dimensional and provide little information on depth, it is impossible to distinguish the uptake in the TGs from that in the nasal mucus. For anatomical evaluation, single photon emission computed tomography/computed tomography (SPECT/CT) images provide superior data because fusion images of SPECT and CT provide three-dimensional anatomy. Therefore, SPECT/CT images are more suitable than planar images for evaluating the precise location of the TGs, which are small, thin, and predominantly overlaying the tori tubarius [1]. Although SPECT/CT scans have been recently performed in salivary gland scintigraphy in some institutions [9], we do not routinely perform SPECT/CT in salivary gland scintigraphy. On the other hand, evaluating thyroid tumors is a good indication of SPECT/CT scans with [^99m^Tc]pertechnetate [10, 11]. In our institution, the nasopharyngeal areas have been scanned in SPECT/CT with [^99m^Tc]pertechnetate performed for the evaluation of thyroid tumors.

[^18^F]fluorodeoxyglucose ([^18^F]FDG) is not used to evaluate the salivary function because [^18^F]FDG uptake in the salivary glands varies considerably among individuals. On the contrary, strong [^18^F]FDG uptake in the tori tubarius is often observed and is considered to be physiological uptake derived from the tubal tonsils [12, 13]. Although the tubal tonsils are likely to exist beneath the TGs [14], it is unknown how much of the [^18^F]FDG uptake in the tori tubarius is derived from the TGs or the tubal tonsils. l-[methyl-^11^C]-methionine ([^11^C]methionine) PET/CT has mainly been used to evaluate brain tumors [15]. [^11^C]methionine physiologically accumulates in the salivary glands [16], and its uptake may help to evaluate the function of the salivary glands [17]. [^11^C]methionine uptake in the salivary glands may reflect protein synthesis in the glands. However, it is unknown how much [^11^C]methionine physiologically accumulates in the TGs.

To clarify the physiological function of the TG, it is necessary to demonstrate whether [^99m^Tc]pertechnetate uptake in the TGs is correlated with that in the other salivary glands, or whether [^18^F]FDG and [^11^C]methionine uptakes in the TGs are correlated with that in the other salivary glands or palatine tonsils. The aim of this study was to retrospectively analyze the uptakes of [^99m^Tc]pertechnetate, [^18^F]FDG, and [^11^C]methionine in the tori tubarius, other salivary glands, and palatine tonsils using [^99m^Tc]pertechnetate SPECT/CT, [^18^F]FDG PET/CT, and [^11^C]methionine PET/CT images, and to confirm whether these three imaging modalities are useful in evaluating TG function.

## Materials and methods

### Patients

This retrospective study design was reviewed and approved by the Institutional Review Board of Saitama Medical University International Medical Center, and the need for written informed consent was waived. We enrolled the patients who underwent [^99m^Tc]pertechnetate scintigraphy including SPECT/CT of the head and neck from January 2009 to October 2020 and the patients who underwent both [^11^C]methionine PET/CT and [^18^F]FDG PET/CT within one month of each other PET/CT scan from January 2008 to December 2016. Patients with a history of Sjogren syndrome, those who had undergone chemotherapy for any malignancy, and those who underwent radiation therapy for malignancy of the head and neck were excluded from the analysis. Furthermore, patients whose imaging quality of FDG PET/CT was inappropriate due to extravasation of [^18^F]FDG and fasting failure before FDG PET/CT scanning were also excluded.

### Imaging protocols

For [^99m^Tc]pertechnetate SPECT/CT, the patients were intravenously administered with 185 MBq of [^99m^Tc]pertechnetate solution, which was obtained from a commercial ^99^Mo-^99m^Tc generator (Ultra-Techne Kow, FUJIFILM Toyama Chemical Co., Ltd., Tokyo, Japan). Prior to imaging, the patients gargled with water to eliminate [^99m^Tc]pertechnetate in the salivary juice of the oral cavity. Planar images and SPECT/CT images were acquired 10 min and 30 min after the injection, respectively. For imaging, we used an integrated SPECT/CT scanner (Symbia Intevo, Siemens, Erlangen, Germany) during 2015–2020 and another SPECT/CT scanner (Symbia T6, Siemens, Erlangen, Germany) during 2009–2014. Both scanners had two detector heads fitted with low-energy, high-resolution collimators. Acquisition of anterior and posterior head-to-chest planar images was followed by SPECT/CT. The SPECT portion of Symbia Intevo was acquired using the following parameters: energy peak, 140 keV with 15% width; acquisition, continuous rotation mode of 180° for each head; projection, a total of 60 over 360° with a dwell time of 20 s/view; reconstruction, ordered subset conjugate gradient minimizer (OSCGM) algorithm with one subset and 48 iterations; voxel size, 2.54 × 2.54 × 2.54 mm; and matrix, 256 × 256. The SPECT portion of Symbia T6 was acquired using the following parameters: energy peak, 140 keV with 15% width; acquisition, continuous rotation mode of 180° for each head; projection, a total of 48 over 360° with a dwell time of 5 s/view; reconstruction, three-dimensional ordered subset expectation maximization (3D-OSEM) algorithm with four subsets and six iterations; voxel size, 4.80 × 4.80 × 4.80 mm; and matrix, 128 × 128. The area that was scanned with SPECT was also scanned using CT with the following parameters: tube voltage, 130 kV; tube current determined by automatic dose modulation; and matrix, 512 × 512.

Before undergoing [^11^C]methionine PET/CT, the patients fasted for at least 4 h. The patients were intravenously administered with 6.0 MBq/kg of [^11^C]methionine solution, which was synthesized on site. The radioactivity of syringes before and after [^11^C]methionine injection was counted, and the injection radioactivity was calculated. PET/CT images were acquired 15 min after [^11^C]methionine injection using a PET/CT system combined with a 6- or 16-slice CT scanner (Biograph 6 or Biograph 16, Siemens, Erlangen, Germany). After brain scanning for 7 min, body scanning from the thigh level to the skull base was performed in 1.5 min per bed position. Images were reconstructed in a 168 × 168 matrix with 3.0 × 3.0 × 5.0 mm voxel size using two-dimensional ordered subset expectation maximization (2D-OSEM) with iteration 3 and subset 8 with a Gaussian filter of 6-mm full width at half maximum. The attenuation correction was performed using transmission CT with the following parameters: matrix, 512 × 512; slice thickness, 5 mm; tube voltage 120 keV; automated tube current modulation (CARD Dose4D, quality reference of 50 mAs); tube rotation, 0.5 s; table speed, 10 mm/rotation; and pitch of 1.0.

[^18^F]FDG PET/CT was performed within one month of [^11^C]methionine PET/CT. The patients fasted for at least 6 h before the test. Blood glucose levels were measured in all patients before [^18^F]FDG administration; no patient had blood glucose levels exceeding 200 mg/dL underwent this test. The patients were intravenously administered with 3.7–4.0 MBq/kg of [^18^F]FDG solution, which was synthesized on site. PET/CT images were acquired 60 min after the injection. After brain scanning for 7 min, body scanning from the thigh level to the skull base was performed in 2 min per bed position. The other PET/CT imaging and reconstruction protocols were identical to those of [^11^C]methionine PET/CT.

### Image analysis

[^99m^Tc]pertechnetate SPECT/CT images were retrospectively reviewed using visual and quantitative methods. Because the TGs are predominantly overlaying the tori tubarius [1], we measured the uptake of the TGs by drawing volumes of interest (VOIs) on the tori tubarius. The three types of images (SPECT, CT, and the fusion images) were visually reviewed to determine whether the uptake in the tori tubarius was higher than that in the background. For quantitative analysis, maximal counts of the tori tubarius, parotid salivary glands (PSGs), submandibular salivary glands (SmSGs), and sublingual salivary glands (SlSGs) were measured by drawing VOIs over SPECT/CT fusion images. As a non-specific reference region, the mean and maximal counts of the posterior neck muscles were also measured using VOIs. The counts of the palatine tonsils were unable to be measured because the counts of the oral cavity were extremely high. The maximal counts of the tori tubarius, PSGs, SmSGs, SlSGs, and the background were divided by the mean counts of the background, and the five ratios were used for comparative analysis. We did not use standardized uptake value (SUV) because our SPECT/CT system did not support calculation of SUV from 2009 to 2015 and also precise injection radioactivity was not measured.

While reviewing the PET/CT images, the maximal radioactivity of [^18^F]FDG and [^11^C]methionine in the tori tubarius, PSGs, SmSGs, SlSGs, and palatine tonsils was measured by drawing VOIs over PET/CT fusion images. The measured radioactivity was expressed as the maximum SUV (SUVmax). The SUV normalizes the measured tissue radioactivity in a VOI by the injected radioactivity and body weight, and it is calculated according to the following formula: SUV = radioactivity concentration in VOI (MBq/mL)/injection radioactivity (MBq/kg body weight).

### Statistical analysis

Quantitative data are expressed as the mean ± SD values. In [^99m^Tc]pertechnetate SPECT/CT analysis, the ratios of the maximal counts of the tori tubarius, three major salivary glands and background to the mean counts of the background were analyzed using one-way analysis of variance (ANOVA) with Dunnett’s multiple comparisons test. In PET/CT analysis, the correlation of SUVmax between the tori tubarius and the other glands was analyzed using Pearson’s correlation analysis. All statistical tests were two-tailed, and *p* values < 0.05 were considered statistically significant. Statistical analyses were conducted using GraphPad Prism 9.0 (GraphPad Software, Inc., San Diego, CA, USA).

## Results

### Patient characteristics

Twelve Japanese patients underwent [^99m^Tc]pertechnetate SPECT/CT from January 2009 to October 2020, and data of all these patients were included in the evaluation. The median age was 62 years (range 46–70 years), and the male-to-female ratio was 9:3.

A total of 218 patients who underwent [^11^C]methionine PET/CT and [^18^F]FDG PET/CT were enrolled. Among them, 85 patients were excluded due to the history of Sjogren syndrome, radiation therapy, and chemotherapy. In addition, three patients were excluded due to extravasation of [^18^F]FDG (two patients) and failure to fast before [^18^F]FDG PET scan (one patient). Finally, data of 130 patients were analyzed. The median age was 55 years (range 0.8–83 years), and the male-to-female ratio was 78:52.

### [^99m^Tc]pertechnetate SPECT findings

**Fig. 1:**
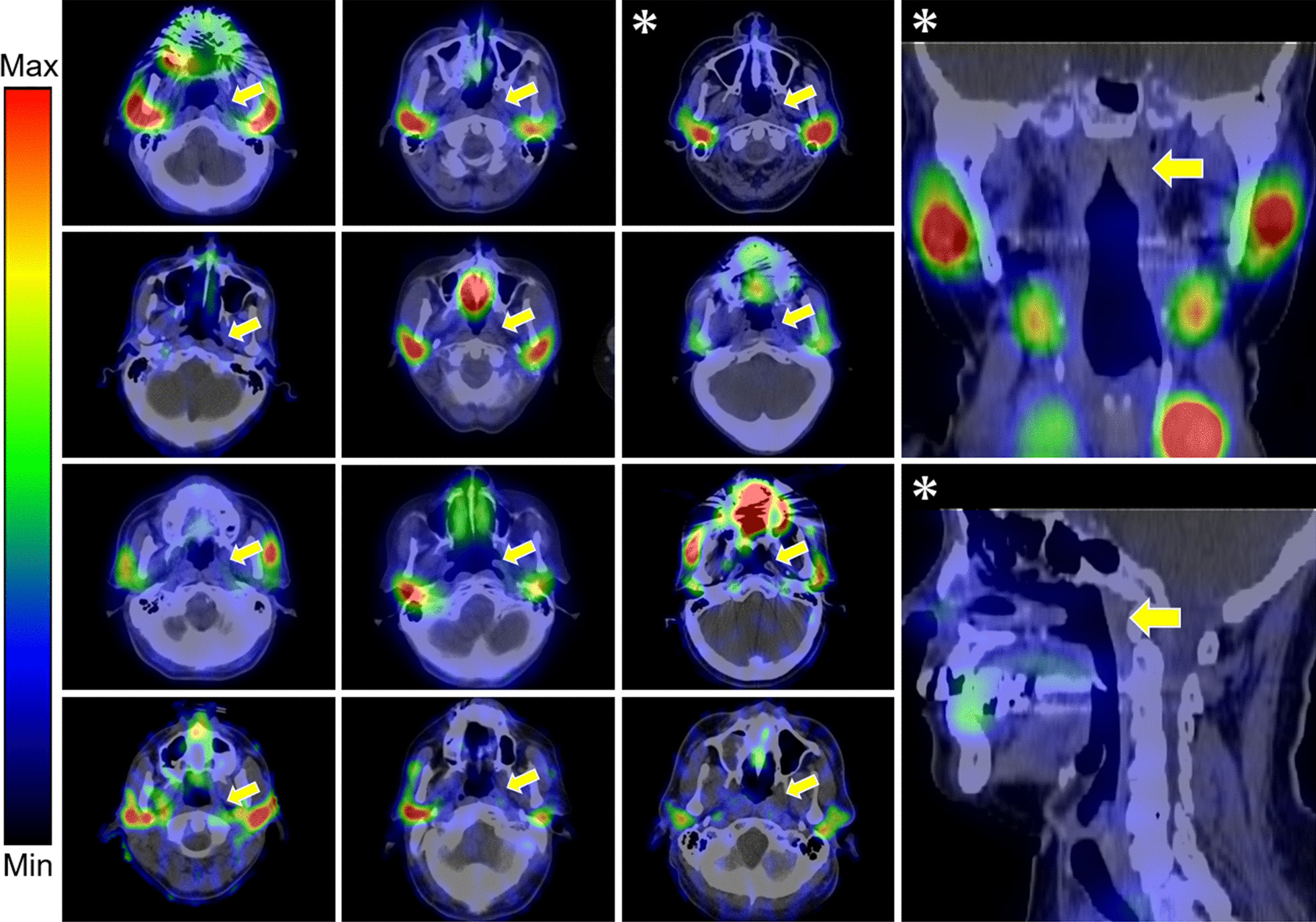
[^99m^Tc]pertechnetate SPECT/CT images of all the patients. Yellow arrows show the torus tubarius. Images marked with asterisks belong to the same patient

**Fig. 2:**
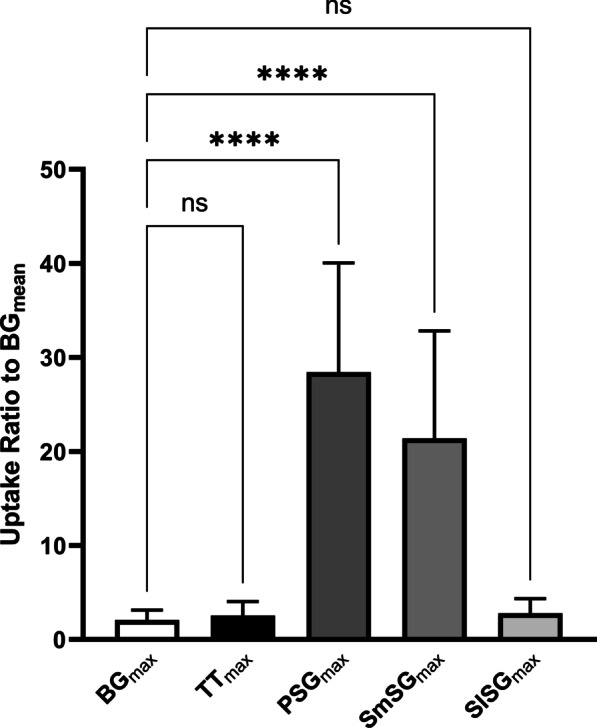
Ratios of maximal counts of [^99m^Tc]pertechnetate in each salivary gland and the background to mean counts of the background. *BG* background, *TT* torus tubarius, *PSG* parotid salivary gland, *SmSG* submandibular salivary gland, *ns* not significant; ****p < 0.0001

Visual analysis showed no significant [^99m^Tc]pertechnetate uptake in the tori tubarius in any of 12 patients (Fig. 1). In the quantitative analysis, the maximal count ratios in the tori tubarius (2.6 ± 1.5) and SlSGs (2.6 ± 1.5) were not significantly higher than those in the background (2.1 ± 1.0, *p* = 0.38), while the maximal count ratios in the PSGs (28.4 ± 11.6) and SmSGs (21.4 ± 11.4) were significantly higher than that in the background (*p* < 0.001) (Fig. 2).

### PET/CT findings of [^18^F]FDG and [^11^C]methionine

**Fig. 3:**
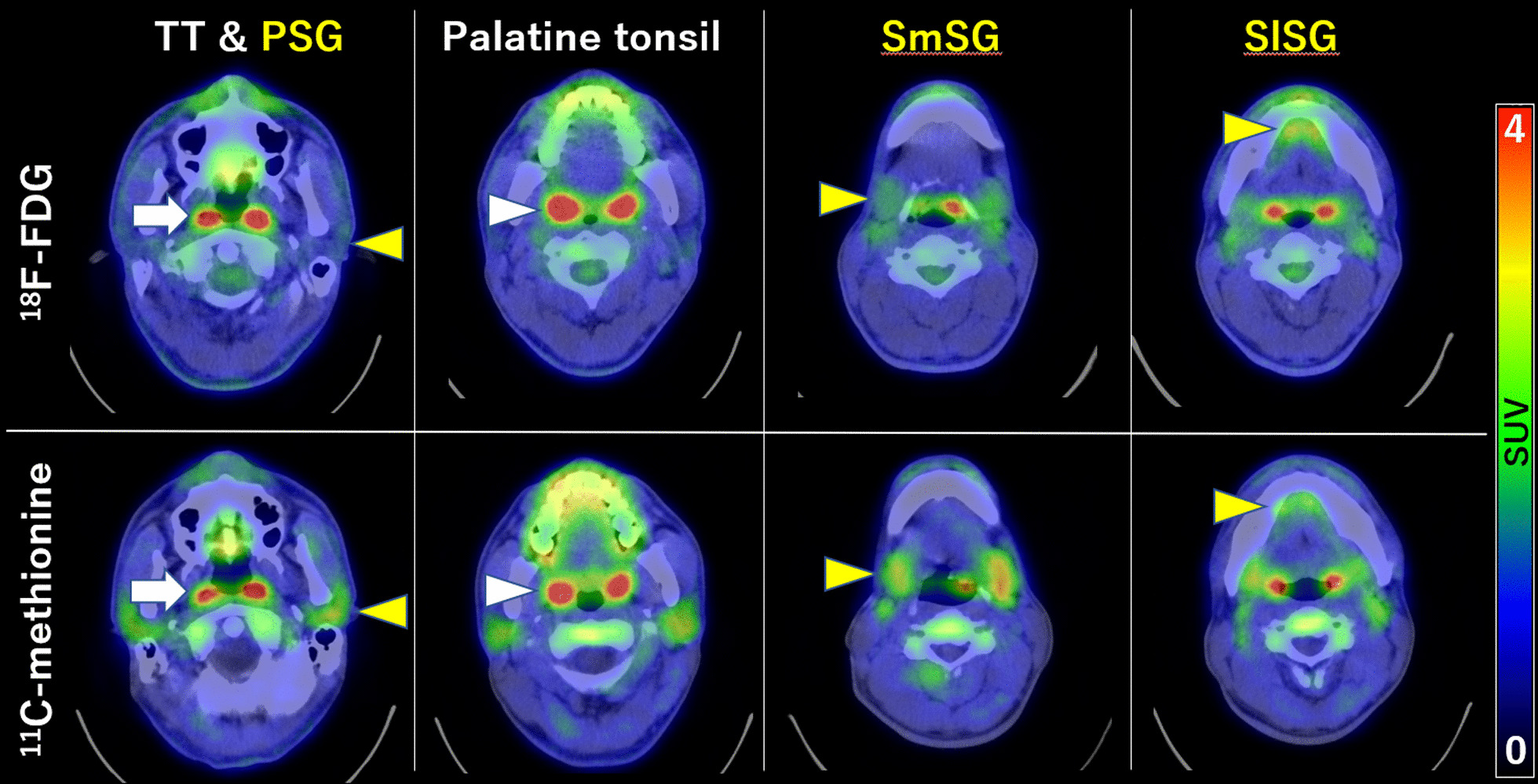
Representative PET/CT images of a 37-year-old male patient. Upper row, [^18^F]FDG PET/CT images; lower row, [^11^C]methionine PET/CT images. TT (tori tubarius, white arrows), Palatine tonsil (white arrowhead), PSG (parotid salivary gland, yellow arrowheads), SmSG, (submandibular salivary gland, yellow arrowheads), and SlSG (sublingual salivary gland, yellow arrowheads)

**Fig. 4:**
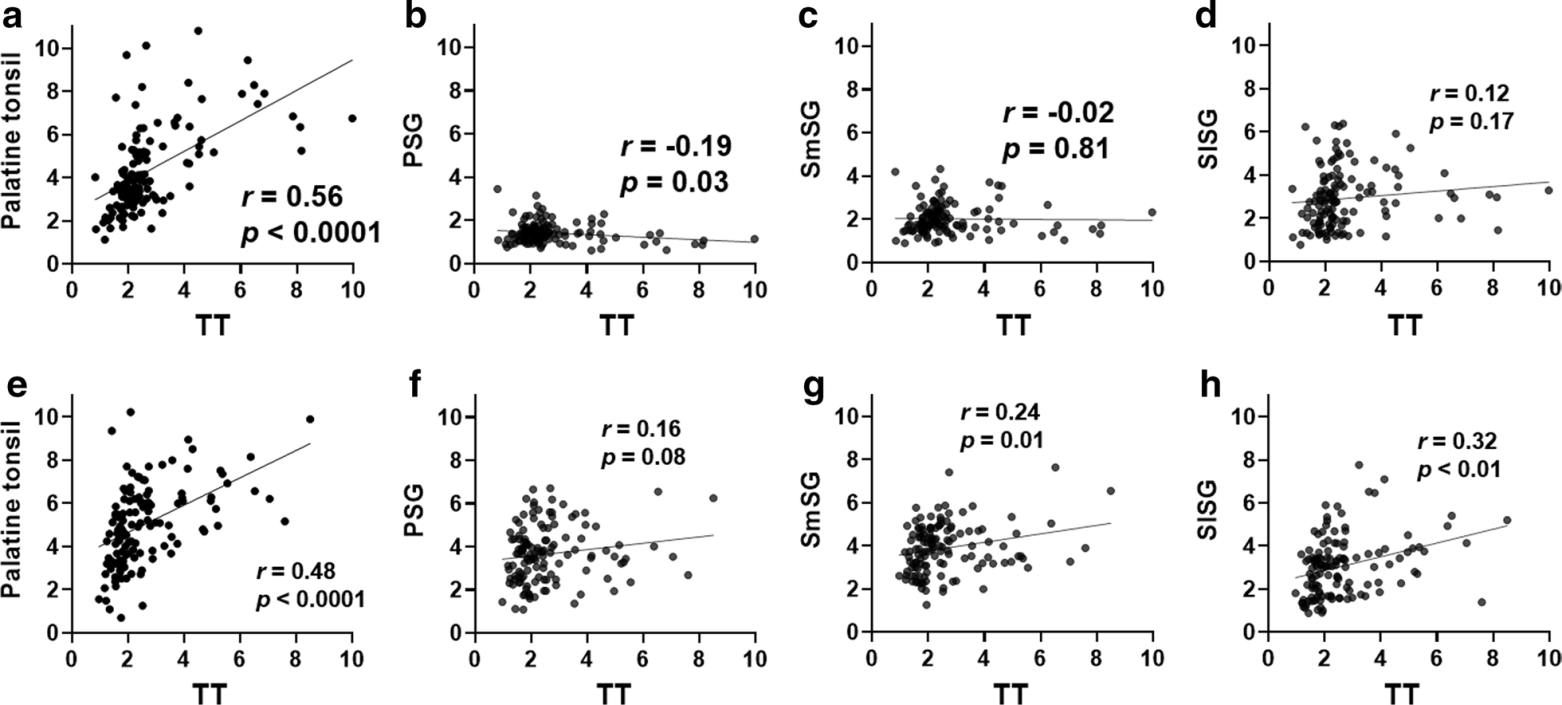
Correlations of SUVmax in the tori tubarius (TT) with that in the palatine tonsil and the other major salivary glands (**a**–**d**, [^18^F]FDG; **e**–**h**, [^11^C]methionine). *PSG* parotid salivary gland, *SmSG* submandibular salivary gland, *SlSG* sublingual salivary gland

The interval between [^18^F]FDG PET/CT and [^11^C]methionine PET/CT was 6.0 ± 7.0 days. Figure 3 shows representative [^18^F]FDG and [^11^C]methionine PET/CT images of a patient’s tori tubarius, each salivary gland, and palatine tonsils. Figure 4 summarizes the results of the uptake correlations between the tori tubarius and the other glands. Both [^18^F]FDG and [^11^C]methionine uptakes in the tori tubarius were positively correlated with that in the palatine tonsils (*r* = 0.56, *p* < 0.0001; *r* = 0.48, *p* < 0.0001, respectively). On the contrary, [^18^F]FDG uptake in the tori tubarius was not positively correlated with that in the PSGs, SmSGs, and SlSGs (*r* = − 0.19, *p* = 0.03; *r* = − 0.02, *p* = 0.81; *r* = 0.12, *p* = 0.17, respectively). [^11^C]methionine uptake in the tori tubarius was positively correlated with that in the SmSGs and SlSGs (*r* = 0.24, *p* = 0.01; *r* = 0.32, *p* < 0.01, respectively). However, [^11^C]methionine uptake in the tori tubarius did not correlate with that in the PSGs (*r* = 0.16, *p* = 0.08).

## Discussion

This study demonstrated that the TGs were invisible on [^99m^Tc]pertechnetate SPECT/CT images, and that [^18^F]FDG and [^11^C]methionine uptakes in the TGs were not undistinguishable from the uptake in the tubal tonsils. To our knowledge, this is the first report evaluating the uptake of radiopharmaceuticals in the TGs other than PSMA ligands.

Our results imply that the TGs were not found until PSMA PET emerged. We did not miss the TGs but had been unable to observe them on [^99m^Tc]pertechnetate images. In addition, we had believed without doubt that [^18^F]FDG and [^11^C]methionine uptakes in the tori tubarius were derived only from the tubal tonsils. If [^99m^Tc]pertechnetate SPECT/CT can help visualize the uptake in the TGs and if the secretion from the TGs is controlled by taste stimulation in the oral cavity, it might have been possible to evaluate their secretory function using the vitamin C stress test. Salivary gland scintigraphy reveals the salivary gland function of both uptake and secretion, and the secretory function can be measured with the vitamin C stress test, which is often used for evaluation of Sjogren syndrome [7, 8]. However, our results suggested that it is not possible to use the stress to evaluate the secretory function of the TGs because the uptake in the TGs itself was not observed. Some reasons behind this invisibility of the TGs on [^99m^Tc]pertechnetate SPECT/CT images are as follows. First, the TGs may express few Na+/K+/2Cl− cotransporters (NKCCls), via which [^99m^Tc]pertechnetate rapidly accumulates in the PSGs and SSGs as substitute for Cl− [18]. [^99m^Tc]pertechnetate imaging does not visualize the sublingual salivary glands [19], whose histological feature is similar to the TGs in that the main type of these glands is mucous type [20]. Therefore, if the invisibility of the sublingual salivary gland on [^99m^Tc]pertechnetate imaging is caused by modest expression of NKCCls, the TGs may also be invisible on images. Second, the spatial resolution of SPECT images may be too low to visualize the TGs. The TGs are thin structures localized on the posterior wall of the nasopharynx [1]. PET has a higher spatial resolution than does SPECT, and PSMA PET/CT can help visualize thin and small tissues such as the TGs. However, PSMA ligands are not released from the salivary glands even with vitamin C stimulation [6]. Therefore, the secretory function cannot be evaluated with PSMA PET/CT. [^18^F]tetrafluoroborate ([^18^F]TFB), a sodium/iodine symporter-target PET tracer, has already been used for PET/CT imaging in healthy humans [21]. [^18^F]TFB strongly accumulates in the salivary glands as well as in the thyroid glands [22]. If [^18^F]TFB is secreted from the major salivary glands, the secretory function of the TGs might also be evaluated using [^18^F]TFB PET.

[^18^F]FDG uptake in the tori tubarius was correlated with that in the palatine tonsils but not with that in the other salivary glands. Although [^11^C]methionine uptake in the tori tubarius was mildly correlated with that in the SmSGs and SlSGs, the correlation was weaker than that between the tori tubarius and the palatine tonsils. These two radiopharmaceuticals are metabolic tracers, and their uptake is not specific to the gland tissues. In particular, the tubal tonsils coexist with the TGs in the tori tubarius, and our results indicated that the uptake of [^18^F]FDG and [^11^C]methionine in the tori tubarius is strongly affected by the tubal tonsils. Before the discovery of the TGs using PSMA PET/CT by Valstar et al., most of the nuclear medicine physicians must have considered that [^18^F]FDG uptake in the tori tubarius is derived only from the tubal tonsils. This is because [^18^F]FDG uptake in the pharyngeal, tubal, palatine, and lingual tonsils, called the Waldeyer ring, often changes in tandem with each other tonsils. Considering that [^18^F]FDG and [^11^C]methionine uptakes in the tori tubarius were positively correlated with that in the palatine tonsil, the uptake of both tracers in the tori tubarius is mainly affected by the tubal tonsils and not by the TGs. Therefore, [^18^F]FDG and [^11^C]methionine uptakes in the tori tubarius cannot be used to evaluate the physiological functions of the TGs. On the contrary, [^11^C]methionine uptake in the tori tubarius was mildly correlated with that in the SmSGs and SlSGs, which suggests that the characteristics of the TGs may be similar to the latter two glands. PSMA ligands specifically accumulate in the salivary glands, and there is no evidence that PSMA ligands accumulate in the tonsils. Therefore, the combinational analysis of PSMA ligands and [^11^C]methionine may be useful to separately assess the glandular tissues and tonsillar tissues.

The definition and nomenclature of the TGs are still debatable [23]. Although Valstar et al. categorized the glands into the salivary glands, the TGs may rather belong to the airway than to the digestive tract. The TGs are located in the nasopharyngeal area where swallowed food does not pass but air from the nose does. Furthermore, Valstar et al. reported that TGs do not produce amylase, an enzyme that degrades of carbohydrates, but produce mucin, which may prevent drying of the nasopharynx. We consider that the entity of the TGs may be closer to “the nasal glands” than the salivary glands, and they may be able to be expressed as “the major nasal glands.” In fact, PSMA ligands accumulate in the minor glands of the nasal cavity [4], which may be called “the minor nasal glands.” Discussion by otolaryngologists and anatomists should be needed to determine the definition or nomenclature of TSs.

The limitation of this study is the lack of a direct comparison of the findings of the three image modalities with those of PSMA PET/CT images. There are no data on PSMA uptake in the TGs in the Japanese population, and the racial differences in parameters such as the size of the TGs were not evaluated. Only a few patients who underwent [^99m^Tc]pertechnetate SPECT/CT, and they did not undergo either [^11^C]methionine or [^18^F]FDG PET/CT.

## Conclusions

We demonstrated that [^99m^Tc]pertechnetate SPECT/CT images did not show significant uptake in the TGs. [^18^F]FDG and [^11^C]methionine uptakes in the tori tubarius were correlated with that in the palatine tonsils, which suggests that the uptake in the tori tubarius is likely to be affected by that in the tubal tonsils. On the contrary, [^18^F]FDG and [^11^C]methionine uptakes in the tori tubarius were little related to that in the other salivary glands. It seems difficult to clarify the physiological function (seromucous fluid-secreting ability) of the TGs using [^99m^Tc]pertechnetate SPECT/CT, [^18^F]FDG, and [^11^C]methionine PET/CT imaging.
